# 

**Published:** 1895-09-28

**Authors:** 


					<ilJrttwnrrroi
CADBURY'S
? COCOA
n'? punty and freedom from alkali." Jg, ? i^izsL? "The TWad Cocoa of Englid, HuabcMn.
Bratthwaite. Absolutely Pure."?The Analyst.
wtfofifbTR7!^71tjfi iyich hospi:
C?;,470- Vo1-xvm' WITH EXTRfl NURSING supplement. ^^5385125:
SEPT. 28th 1895 [Registered at the Goneral Post Offloe aa a Newspaper for Monthly Parts, 6d.; post free, 8d.
* transmission in the United Kingdom and Abroad.] Ditto per Annnm.8a.6d., post free.

				

